# Predicting Workers’ Stress: Application of a High-Performance Algorithm Using Working-Style Characteristics

**DOI:** 10.2196/55840

**Published:** 2024-08-02

**Authors:** Hiroki Iwamoto, Saki Nakano, Ryotaro Tajima, Ryo Kiguchi, Yuki Yoshida, Yoshitake Kitanishi, Yasunori Aoki

**Affiliations:** 1 Shionogi & Co., Ltd. Osaka Japan; 2 Department of Psychiatry Nippon Life Hospital Osaka Japan

**Keywords:** high-performance algorithm, Japan, questionnaire, stress prediction model, teleworking, wearable device

## Abstract

**Background:**

Work characteristics, such as teleworking rate, have been studied in relation to stress. However, the use of work-related data to improve a high-performance stress prediction model that suits an individual’s lifestyle has not been evaluated.

**Objective:**

This study aims to develop a novel, high-performance algorithm to predict an employee’s stress among a group of employees with similar working characteristics.

**Methods:**

This prospective observational study evaluated participants’ responses to web‑based questionnaires, including attendance records and data collected using a wearable device. Data spanning 12 weeks (between January 17, 2022, and April 10, 2022) were collected from 194 Shionogi Group employees. Participants wore the Fitbit Charge 4 wearable device, which collected data on daily sleep, activity, and heart rate. Daily work shift data included details of working hours. Weekly questionnaire responses included the K6 questionnaire for depression/anxiety, a behavioral questionnaire, and the number of days lunch was missed. The proposed prediction model used a neighborhood cluster (N=20) with working-style characteristics similar to those of the prediction target person. Data from the previous week predicted stress levels the following week. Three models were compared by selecting appropriate training data: (1) single model, (2) proposed method 1, and (3) proposed method 2. Shapley Additive Explanations (SHAP) were calculated for the top 10 extracted features from the Extreme Gradient Boosting (XGBoost) model to evaluate the amount and contribution direction categorized by teleworking rates (mean): low: <0.2 (more than 4 days/week in office), middle: 0.2 to <0.6 (2 to 4 days/week in office), and high: ≥0.6 (less than 2 days/week in office).

**Results:**

Data from 190 participants were used, with a teleworking rate ranging from 0% to 79%. The area under the curve (AUC) of the proposed method 2 was 0.84 (true positive vs false positive: 0.77 vs 0.26). Among participants with low teleworking rates, most features extracted were related to sleep, followed by activity and work. Among participants with high teleworking rates, most features were related to activity, followed by sleep and work. SHAP analysis showed that for participants with high teleworking rates, skipping lunch, working more/less than scheduled, higher fluctuations in heart rate, and lower mean sleep duration contributed to stress. In participants with low teleworking rates, coming too early or late to work (before/after 9 AM), a higher/lower than mean heart rate, lower fluctuations in heart rate, and burning more/fewer calories than normal contributed to stress.

**Conclusions:**

Forming a neighborhood cluster with similar working styles based on teleworking rates and using it as training data improved the prediction performance. The validity of the neighborhood cluster approach is indicated by differences in the contributing features and their contribution directions among teleworking levels.

**Trial Registration:**

UMIN UMIN000046394; https://www.umin.ac.jp/ctr/index.htm

## Introduction

Stress is an external or internal stimulus that produces a compensatory biological response that can trigger or aggravate many diseases or pathological conditions [1]. Notably, the stress-depression association requires recognizing the effects of context and personal characteristics on the existence of stressors and understanding the progressive and dynamic relationship between stress and depression over time [2]. This is important because depression remains a major social issue [3] with a high relapse rate, prolonged duration of illness [4], and high socioeconomic impact [5]. The duration of untreated depression is associated with worse outcomes [6]. The annual national cost of major depressive disorder among adults aged ≥20 years in Japan in 2008 was approximately US $11 billion, including US $6.9 billion in workplace‑associated expenses [5].

Detecting and targeting depression before a formal diagnosis can serve as an early countermeasure to depression. Therefore, detecting stress in advance is vital because stress is a factor that triggers depression and increases the risk of relapse [2]. Companies are placing an ever‑increasing emphasis on their employees’ mental health, including their experience of stress, as an important topic to address. According to the Japanese Ministry of Health, Labour and Welfare (2021), the proportion of companies with workers taking temporary leave or retiring due to mental health conditions has increased from 9.2% in 2020 to 10.1% in 2021 [7]. Furthermore, about 40% of companies in Japan reported worsening employee mental health due to the COVID-19 pandemic [8]. Therefore, in response to this growing need, the proportion of companies conducting stress checks on their employees has increased from 62.7% in 2020 to 65.2% in 2021 in Japan [7].

One approach is to develop stress prediction models using data related to stress collected by wearable devices that measure parameters such as heart rate variability [9], physical activity [10], and sleep [11], as well as through questionnaire responses that provide insights into physical activity [12] (eg, outings), absenteeism (failure to report for scheduled work), and the number of times lunch is missed [13]. However, these data are affected by working style such as teleworking habits (eg, remote working).

To the best of our knowledge, there is no study taking teleworking habits into account for stress prediction even though the relationship between teleworking and stress has been studied. Teleworking/telecommuting can have an impact on mental health [14,15]. However, stress is dependent not only on the environment but also on an individual’s attributes [16,17]. Moreover, stress parameters [9,18,19] can be influenced by various other factors. Consequently, a few studies on stress detection have used a personalized model-based approach [20-22].

The objective of this study was to develop a novel, high-performance stress prediction algorithm using working data focusing on employees’ teleworking habits.

## Methods

### Study Design

This prospective observational study (UMIN000046394) evaluated participants’ responses to web-based questionnaires, including attendance records and data collected via a wearable device. The data were used to develop a high-performance stress prediction algorithm based on working-style characteristics similar to those of the prediction target person among the participants. Data spanning 12 weeks were collected for each employee from January 17, 2022, to April 10, 2022.

### Ethical Considerations

Informed consent was obtained from employees using a web-based consent form. This study was approved by the Research and Ethics Committee of Shionogi & Co., Ltd (EP21-13) and the MINS Institutional Review Board (210238), a specified nonprofit organization. The study was conducted in compliance with the ethical guidelines for medical and health research involving human participants and in accordance with the ethical principles of the Declaration of Helsinki. To deidentify the participants, age and sex data were not collected.

### Recruitment

This study enrolled 194 employees of the Shionogi Group working in Osaka, Japan. Participants who rarely teleworked included sales or research employees, and those who frequently teleworked included clerical employees. Notably, neither 100% teleworking nor teleworking other than working from home was permitted for Shionogi Group employees. The teleworking rate was calculated as the number of days an employee worked from home during the 12 weeks divided by the number of days an employee worked during the 12 weeks.

The participants, who were from different departments, worked during standard working hours (9 AM to 5 AM Monday to Friday); however, given the anticipated flexible time system for data collection, participants could decide their working hours each day and enter work start and end times into the attendance management system in advance. Night shift workers were not included in this study, and while there was a certain degree of flexibility in work hours, daytime workers were encouraged not to shift their work hours too far from the standard workday except when necessary. There were no exclusion criteria other than working time and region (daytime employee, working in Osaka), thereby reducing enrollment bias.

### Data Collection

Daily data collected from the Fitbit Charge4 wearable device worn for 12 weeks (Fitbit LLC) included sleep data recorded daily (sleep duration, sleep efficiency, sleep initiation, and end time), activity data recorded every 15 min (number of steps taken, distance moved, number of floors climbed or descended, and calories burned), and heart rate per minute. Daily work shift data collected included working hours, scheduled work start and end times, scheduled hours of work, work from home (yes/no), and absence from work/leave taken (yes/no).

Weekly web-based questionnaire responses included the K6 questionnaire [23,24], which measures 6 common symptoms of depression and anxiety, each rated on a scale between 0 and 4 (0=never, 1=a little, 2=sometimes, 3=most often, and 4=at all times). The total score was the sum of the responses to each question (ranging from 0 to 24), the behavioral questionnaire (number of outings, such as commuting and social outings), and the number of days lunch was missed. We selected the latter 2 parameters based on the premise that the number of outings is an alternative index for exercise habits [12]. Outings could also be used as an alternative index for UV exposure, which is reported to be related to mental health [25,26], and skipping lunch is reported to be related to stress [13].

### Proposed Prediction Model

#### Step 1: Extract the Neighborhood Cluster

The participants were arranged in ascending order based on their teleworking rate, with each participant serving as a prediction target person. To homogenize the training data background, a group of participants whose working style/work characteristics were similar to those of the prediction target person were extracted and labeled as the neighborhood cluster. This neighborhood cluster included participants with the top 20 nearest teleworking rates (for the training data) from the prediction target person. In some instances, when the size of the neighborhood cluster was greater than 20 because of the same ranking on the boundary, participants on the boundary were randomly sampled to include only 20 participants.

#### Step 2: Create an Individual Model to Predict Stress

**Figure 1:**
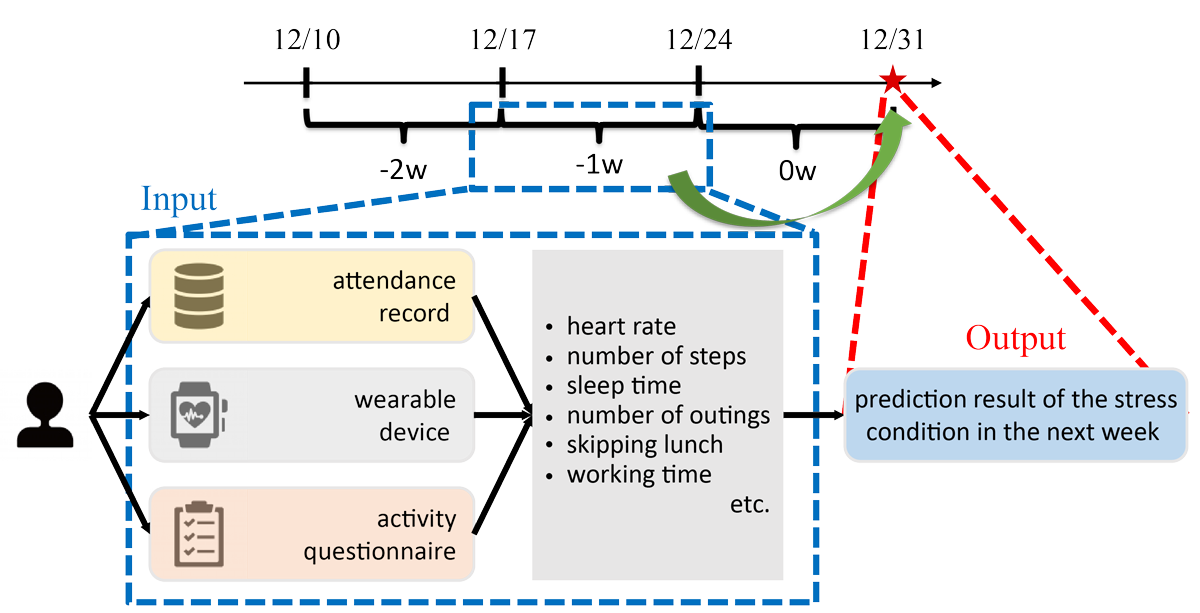
Prediction model. Data collected within a term shown by a blue dashed-line box are input to the prediction model, and the stress state (negative/positive) at the timepoint shown by a red star is predicted.

The selected neighborhood cluster was subsequently used to train a prediction model for each prediction target person, meaning that an “n” number of different prediction models was created for the “n” number of targets to be included in this analysis. Using the neighborhood cluster data extracted in Step 1, a model was created that was individually optimized for the prediction target person. Data from the previous week were used to predict the stress level in the following week using this individual model. Although data for 12 weeks were collected, only the data for 11 weeks were used in the model because the data before week 1 (–1 week) were not collected to use the first-week data in the model (Figure 1).

**Figure 2:**
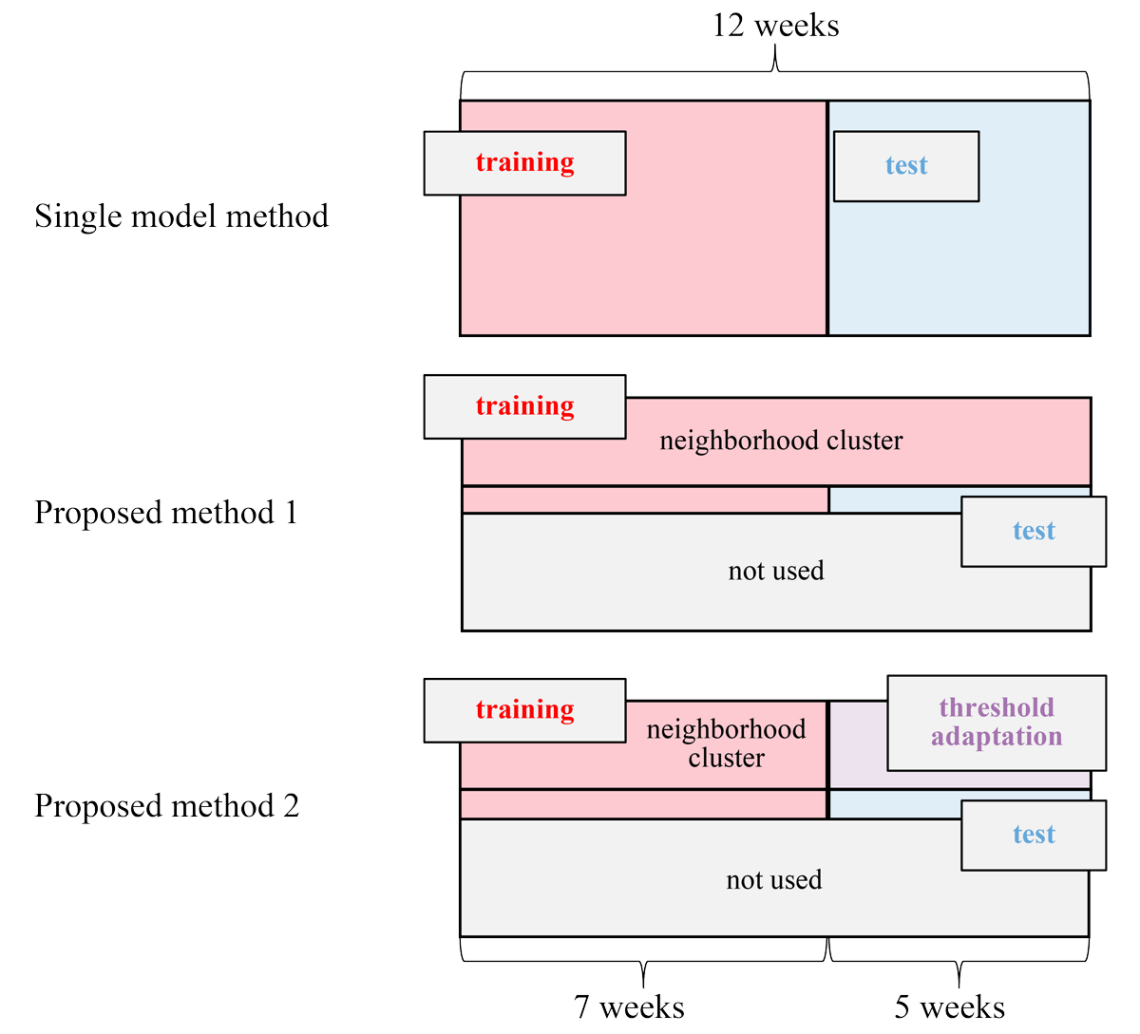
Twelve-week data split for comparison of the three methods.

The 12-week data were split into training and test data for the 3 models. The training data comprised all 12-week data of the neighborhood cluster plus data from the first 7 weeks for the prediction target person. The test data comprised the last 5 weeks of data from the prediction target person (Figure 2).

### Analysis Method

#### Sample Size

Considering the feasibility of an exploratory evaluation, the number of study participants was set to 150. However, the proportion of people with mental illness at the Shionogi Group was estimated to be between 7% and 10%, and the expected participation of approximately 10 patients with mental illness was based on this value. In general, too few mental illness cases lead to failure of analysis, whereas too many mental illness cases (>10%) do not appropriately reflect the population. As a screening method, we collected a stress check questionnaire when obtaining informed consent. However, as the number of mental illness cases was within the expected range of 7% to 10%, a formal screening was not performed. A total of 2037 weeks of data were evaluated. Data were evaluated weekly, and the mean (SD) was calculated from each participant’s weekly data. The mean was omitted only when data were missing for the entire 7 days of the week, and the SD was omitted only when data were missing for ≥6 days of the week (unbiased SD required 2 or more data points). The K6 questionnaire scores representing the stress index [23] were converted into binary objective variables (negative=K6: 0-4 [class 1]; positive=K6: 5-8 [class 2], K6: 9-12 [class 3], and K6: ≥13 [class 4]).

#### Model Training Details

The analysis was performed using Python (version 3.8.0; Python Software Foundation) and PyCaret (version 2.3.10). The Extreme Gradient Boosting (XGBoost) hyperparameters were set as follows (common in all cases): max_depth=6, learning_rate=0.3, and n_estimators=100. These hyperparameter values are the default configuration of PyCaret, and a hyperparameter search was not performed. The 3 models were compared, which included threshold adaptation. The single model used the first 7 weeks as training data and the latter 5 weeks as test data for all participants. Proposed method 1 used 12-week data of the neighborhood cluster plus the first 7-week data of the prediction target person as training data and the latter 5-week data of the prediction target person as test data. Both methods used a fixed threshold of 0.5 (the default threshold of XGBoost); an output of the stress prediction model above this threshold indicated high levels of stress. Proposed method 2 used 7-week data of the neighborhood cluster and the prediction target person as training data, the latter 5-week data of the prediction target person as test data, and the latter 5‑week of the neighborhood cluster for threshold adaptation. The explanatory variables are the 50 features shown in Multimedia Appendix 1, and the object variable is the binarized stress score.

The threshold was adjusted such that the true positive (TP) rate was >0.8 using the threshold adaptation data. A value of 0.8 was the practically required TP rate. Of note, there was no guarantee that the TP rate would be >0.8 in the test data because the threshold was not adjusted for test data. The prediction threshold was adjusted such that the TP rate increased to >0.8, with the lowest false positive (FP) rate. Notably, determining the TP rate is more important than determining the FP rate to ensure early depression countermeasures. Thus, by setting the value to 0.8, we could predict as many positives as possible. The area under the receiver operating characteristic curve (AUROC) was used to measure the performance of the models.

#### Data Exclusion

A total of 190 individual models were created, as 2 participants discontinued the study, and data from 2 other participants were missing in the latter 5 weeks and were not included in the test data. However, the data of the latter 2 participants were available for the first 7 weeks and were thus included in the training data (Figure 2).

### Procedure for Checking Feature Contribution

We selected figures to report the absolute amount of feature contribution and feature contribution variability between teleworking rates. Feature importance for the prediction was evaluated for each individual model using XGBoost [27,28], and the top 10 features were identified. High feature importance was defined as the factor (50 variables shown in Multimedia Appendix 1) with a high contribution (influence) to the prediction. Feature importance was defined as a score calculated based on the reduction in the objective function related to heterogeneity (sum of squared residuals for continuous variables and the Gini index for categorical variables) achieved by splitting the feature value when creating decision trees (Multimedia Appendix 2) [28].

Thereafter, the individual model was divided into 3 levels stratified by the teleworking rate, and the top 10 feature values for each level were extracted. Finally, Shapley Additive Explanations (SHAP) [29] were calculated for the top 10 extracted features to evaluate their impact and contribution direction, stratified by 3 levels of teleworking rates, as follows: (1) low: <0.2 (mean of >4 days per week in office), (2) middle: 0.2 to <0.6 (mean of 2-4 days per week in office), and (3) high: ≥0.6 (mean of <2 days per week in office). The absolute value of SHAP represents the contribution amount, while its positive or negative direction on the y-axis represents the contribution direction.

The contribution direction and impact of features were based on “covariance of features and SHAP” divided by “SD of features.” Any positive deviation from 0 on the y-axis was considered to positively impact stress, and any negative deviation was considered to negatively impact stress.

## Results

### Overall Findings

**Figure 3:**
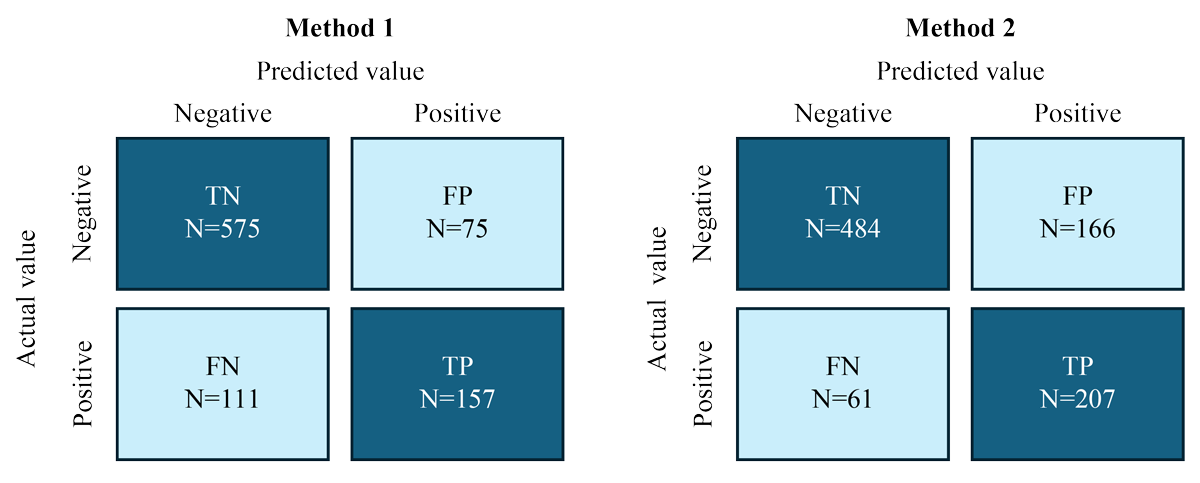
Confusion matrix for methods 1 and 2. “N” represents the total number of target classes. FN: false negative; FP: false positive; TN: true negative; TP: true positive.

Data from 190/194 (97.9%) participants were included to develop high-performance stress prediction algorithms; 2 participants discontinued the study, and data from 2 other participants were included only in the training set. The teleworking rate of the employees ranged between 0% and 79%. The prediction results of the individual models were integrated for all participants using proposed methods 1 and 2 and compared with the results of the single model. Although the proposed methods improved the prediction performance, the AUC was similar for proposed methods 1 and 2. The AUC was the highest for proposed method 1, at 0.85 (TP vs FP: 0.59 vs 0.12), followed by proposed method 2, at 0.84 (TP vs FP: 0.77 vs 0.26) and the single model method, at 0.76 (TP vs FP: 0.42 vs 0.12) (Table 1). The confusion matrix for methods 1 and 2 is presented in Figure 3.

**Table 1:** Comparison of prediction results of the single model method and proposed methods 1 and 2.

Performance metric	Single model	Proposed method 1	Proposed method 2
True positive rate	0.42	0.59	0.77
False positive rate	0.12	0.12	0.26
AUROC^a^	0.76	0.85	0.84

^a^AUROC: area under the receiver operating characteristic curve.

### Feature Importance Analysis

The top 10 features with the highest mean feature importance ranking for each of the 3 teleworking levels are presented in Multimedia Appendix 2. These 10 features were divided into 3 categories: activity (red), work (green), and sleep (blue). They were then tabulated by teleworking levels, with 43.2% (n=82) at the low level, 36.3% (n=69) at the middle level, and 20.5% (n=39) at the high level. Among the participants with a low teleworking rate, most features were related to sleep, followed by activity and work. Among the participants with high teleworking rates, most features were related to activity, followed by sleep and work.

### Analysis of Feature Contribution Direction Based on SHAP

**Figure 4:**
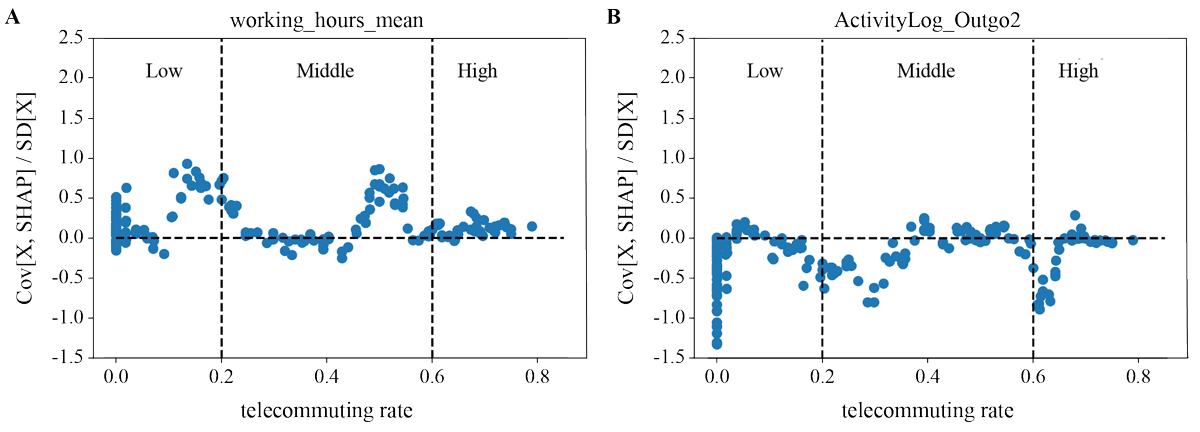
Analysis of the contribution direction of (A) working hours and (B) activity categorized by teleworking/telecommuting rates based on Shapley Additive Explanations (SHAP).

The contribution direction of each individual model for the top 10 extracted features was examined at each level. Although many features were evaluated, only those with interesting suggestions have been reported. Middle and low teleworking rates and longer working hours contributed to higher stress levels (Figure 4A). Irrespective of the teleworking rate, lower activity contributed to higher stress levels (Figure 4B).

**Figure 5:**
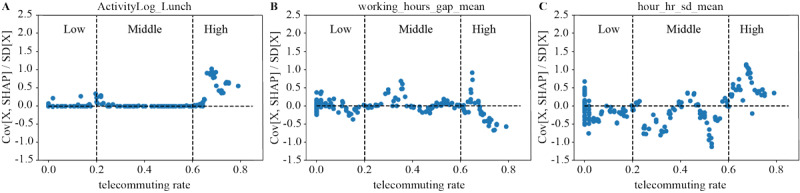
Analysis of the contribution direction of (A) skipping lunch, (B) working hour gap (working more or less than scheduled hours), and (C) heart rate categorized by teleworking/telecommuting rates based on Shapley Additive Explanations (SHAP).

Participants with a high teleworking rate who skipped lunch more often had higher stress levels than those with low or middle teleworking rates. Interestingly, skipping lunch did not contribute to stress prediction in participants with middle and low teleworking rates (Figure 5A). Working more or less than scheduled hours (high variation in the working hour gap) contributed to stress, especially for those with high teleworking rates (Figure 5B). Low fluctuations in heart rate (SD of the heart rate) contributed to stress, particularly for those with middle or low teleworking rates. However, high fluctuations in heart rate were a noticeable contributor to stress in those with a high teleworking rate (Figure 5C).

**Figure 6:**
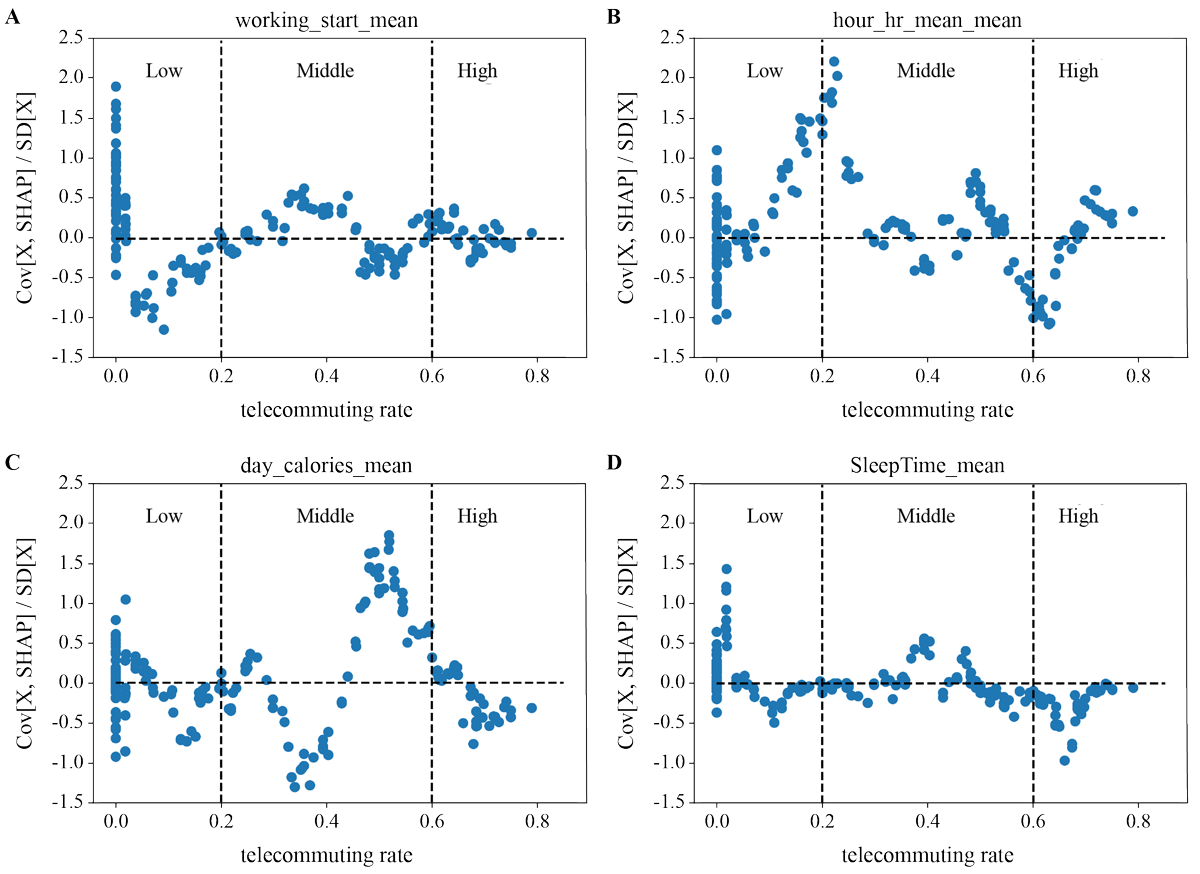
Analysis of the contribution direction of (A) mean work start time, (B) mean heart rate, (C) daily calories burned, and (D) sleep duration categorized by teleworking/telecommuting rates based on Shapley Additive Explanations (SHAP).

In participants with low teleworking rates, being late for work or coming to work too early contributed to stress. Although the variation was lower, a similar trend was observed for participants with high and middle teleworking rates (Figure 6A). Having a heart rate higher or lower than the mean heart rate contributed to stress in participants with low teleworking rates. Although the variation was lower, a similar trend was observed for participants with high and middle teleworking rates (Figure 6B). Burning more or fewer calories than the mean calorie burned contributed to stress in participants with middle and low teleworking rates. Moreover, burning less than normal calories was a noticeable contributor to stress in participants with high teleworking rates (Figure 6C). In participants with a low teleworking rate, a longer mean sleep duration contributed to stress, whereas in those with a high teleworking rate, a lower mean sleep duration was a noticeable contributor to stress (Figure 6D).

## Discussion

### Principal Findings

This study evaluated a novel, high-performance stress prediction algorithm that uses data from employees to extract neighborhood data on working styles or work characteristics similar to those of the target person. The prediction performance of both proposed methods was markedly improved compared with that of the single model (baseline). A good stress prediction performance was achieved—the AUC was the highest for proposed method 1 (0.85), followed by proposed method 2 (0.84) and the single model method (0.76). The level of predictive performance achieved by the proposed models suggested the benefits of narrowing the training data (by using neighborhood data) based on the teleworking rate.

In a stress detection study performed by Tazarv et al [30], per-individual models were reported to outperform single models; however, the approach required many data points (approximately 300 times/month) from participants. Therefore, by selecting a neighborhood cluster, the burden on participants was reduced. This approach alleviated user burden by reducing the number of label requests to 7 data points for the prediction target person. Because previous studies [20-22] did not narrow the training data based on work style/characteristics, it is possible to improve their prediction performance by incorporating this approach.

The results showed that personal data from the prediction target person are important (particularly in terms of measuring the change from baseline) because proposed method 2 showed prediction performance similar to that of proposed method 1. There was almost no difference in the AUC between proposed methods 1 and 2, suggesting that intraindividual fluctuation is a major stressor as the participants’ own data contributed greatly to the performance prediction rather than the neighborhood cluster data. Thus, personal data from the prediction target person are important because a reduction in the neighborhood cluster’s training data to 5 weeks caused no noticeable performance deterioration. Furthermore, the validity of using individual models is supported by the fact that there are differences in the feature contribution depending on the teleworking level, and the direction of the contribution changes within each level.

For participants with low teleworking rates, most features were related to sleep, followed by activity and work. This indicates that the contribution of activity may be lower when working from the office (low teleworking rates) than at other teleworking levels because it is difficult to discriminate between regular activity and activity due to commuting. For participants with high teleworking rates, most features were related to activity, followed by sleep and work. This implies that in a teleworking environment (such as at home), baseline activity levels are consciously assumed to be low and easier to discern than sleep and work.

The results of SHAP suggest that some features are consistent with intuition and common sense, contributing to its validity. Longer working hours among participants with middle and low teleworking rates were a marker of high stress. Low activity, irrespective of the number of days worked from the office per week, was a marker of high stress. Additionally, some features showed changes in the contribution direction within teleworking levels, suggesting the validity of the proposed method for modeling a small group of participants.

Several features characteristic of the high teleworking group, which tended to have the same working style among individuals but in a completely different working environment, were identified. Skipping lunch while working from home was likely to be a marker of stress. This could also be attributed to the fact that with a high degree of freedom, a person is more likely to skip meals. In addition, biological information, such as skipping meals/hunger, is not as easily discernible by employees as activity, which is presumed to be low while teleworking. Additionally, working more or less than the scheduled hours contributed to stress, especially among those with a high teleworking rate. This observation suggested that arriving late or leaving early for appointments may be detected as a sign of stress, likely due to the high psychological hurdles for arriving late or leaving early, especially among those working from the office. We believe that psychological hurdles are fewer when working from home, possibly due to the higher degree of flexibility in using the provided working hours.

Additionally, lower fluctuations in heart rate were found to contribute to stress, especially in participants with middle and low teleworking rates. However, a higher fluctuation in heart rate was a noticeable contributor to stress in those with a high teleworking rate. Although it is known that the lower the fluctuations in heart rate, the greater the stress [9], contradictory results were noted in the high teleworking group. The autonomic nervous system, which consists of sympathetic and parasympathetic nerves, regulates heart rate. During a fight or flight response (work stress or activity in the contemporary sense), sympathetic nerves increase the heart rate. On the other hand, during the rest and digest state (relaxing or inactivity), the parasympathetic nerves dominate and decrease heart rate. It is assumed that sympathetic activation is dominant while working from the office and parasympathetic activation is dominant while teleworking [18]. The low fluctuations in heart rate associated with high stress levels in the low and middle teleworking groups could be attributed to sustained sympathetic dominance with less time to relax while working from the office. Similarly, high fluctuations in heart rate associated with high stress levels in the high teleworking group could be attributed to temporal activation of sympathetic nerves while performing a difficult task, despite the parasympathetic predominance of the baseline state. Additionally, a lower mean sleep duration among participants with a high teleworking rate was a marker of stress in this study. This result is important because we expect that a person should get sufficient sleep when working from home.

Similarly, several features characteristic of the low teleworking group were identified. Coming late or too early to work was identified as a marker of stress among those with a low teleworking rate. These observations suggested that coming too early may correlate with long working hours and coming late may correlate with decreased engagement. Moreover, having a higher or lower than mean heart rate was found to be a marker of stress in those with a low teleworking rate. This suggests that in terms of heart rate, an individual may respond differently to stress while working from the office, according to the baseline state of the autonomic nervous system with sympathetic or parasympathetic dominance. Moreover, the variability in the contribution of calories burned was high among those with middle and low teleworking rates. Burning more or fewer calories than normal among participants with middle and low teleworking rates was a marker of stress and could be attributed to the individual’s unique response.

### Limitations

The data used in this study (ie, wearable device, questionnaire, and attendance data) were affected by working style and various other factors. If the target population were to change, the results may be different from those obtained in this study. Moreover, age-related comorbidities and lifestyle changes were not considered in the modeling, which can impact the outcome. In this study, we created a neighborhood cluster based on the teleworking rate. Therefore, it can only be applied to people who are allowed to telework. The “neighborhood cluster” in this study was assumed to be a “cluster with similar working style.” For practical purposes, it is conceivable that working styles differ greatly, even if the teleworking rate is similar (eg, when data are obtained from multiple companies). Moreover, responses to the questionnaires, including the K6 questionnaire, were subjective for the participants and not necessarily accurate. Furthermore, feature importance and SHAP only quantify the degree to which the machine learning model uses the features for prediction but do not consider whether the model makes predictions with high accuracy. Thus, although the tendency to judge that stress is high when the value of a feature is large is correct, it cannot be confirmed that “stress increases when the value of a feature is large.” Finally, because teleworking outside of working from home was not allowed in the Shionogi Group, a certain degree of participant bias may exist because certain job functions were not permitted to telework. Therefore, the results of this study might not be reproducible when targeting other forms of teleworking.

### Conclusion

Prediction performance was improved by forming a cluster (neighborhood cluster) with similar working styles based on the teleworking rate and using it as the training data. The validity of the neighborhood cluster approach is indicated by differences in the contributing features and their contribution directions among teleworking levels. Further studies are required to evaluate and improve the proposed method using data obtained from employees of different companies. This methodology can improve existing stress detection methods by incorporating the idea of this research and narrowing the training data (ie, neighborhood cluster extraction based on the teleworking rate). This study paves the way for employers to consider and support timely and appropriate interventions for people predicted to experience high stress levels.

## Appendix

Multimedia Appendix 1 Variables evaluated to deduce the feature importance.

Multimedia Appendix 2 Top 10 features with the highest mean feature importance ranking categorized into three levels of teleworking rates using Extreme Gradient Boosting (XGBoost). Features related to activity are in red, features related to work are in green, and features related to sleep are in blue.

## Abbreviations

AUROC: area under the receiver operating characteristic curve
FP: false positive
SHAP: Shapley Additive Explanations
TP: true positive
XGBoost: Extreme Gradient Boosting
