# Supplementary material for: Predicting Workers’ Stress: Application of a High-Performance Algorithm Using Working-Style Characteristics
Source: JMIR AI. 2024 Aug 2;3:e55840. doi: 10.2196/55840 (PMC11329844; doi:10.2196/55840)
Supplement: Multimedia Appendix 1 [file ai_v3i1e55840_app1.docx]

**Table S1 Multimedia Appendix 1.** Variables evaluated to deduce the feature importance.

|  | **Variable Name** | **Comment** | **Processing** |
| --- | --- | --- | --- |
| **1** | ActivityLog_Lunch | Behavioral log data (lunch) | Number of missed lunches within 1 week |
| **2** | ActivityLog_Outgo1 | Behavioral log data (including outgoing) | Number of days going outside within 1 week (including commuting) |
| **3** | ActivityLog_Outgo2 | Behavioral log data (outgoing: not discharged) | Number of days going outside within 1 week (not including commuting) |
| **4** | Work_home_rate | Home working rate | Rate of work at home during the most recent week (including the assessment time point) at the time of assessment (only on workdays included) |
| **5** | Working_hours_mean | Mean working hours | Mean work hours in the most recent week (including the evaluation time point) at the evaluation time point (only for workdays). The data were compiled by application time rather than by stationary time. One-hour break is excluded in the case of overtime of 6 hours |
| **6** | Working_hours_sd | SD of working hours | Variability in work hours during the most recent week (including assessment time point) at the assessment time point (SD). Units are minutes. Compilation based on application time rather than on the time spent in the stationary period. One-hour break is excluded in the case of overtime of 6 hours |
| **7** | Working_start_mean | Mean work start time | Mean starting time of work in the last week (including the evaluation time point) at the time of assessment (only on workdays; tabulated by submission time rather than by check-in time) |
| **8** | Working_start_sd | SD of the starting time of work | Variation (SD) in work initiation times during the most recent week (including assessment time point) at the assessment time point. Units are minutes. (only on workdays; tabulated by submission time rather than by check-in time) |
| **9** | Working_end_mean | Mean work completion time | Mean working hours in the most recent week (including the evaluation time point) at the time of assessment (only on workdays; tabulated by submission time rather than by check-out time) |
| **10** | Working_end_sd | SD of work completion time | Variation (SD) in work completion times in the most recent week (including the assessment time point) at the assessment time point. Units are minutes (only on workdays; tabulated by submission time rather than by check-out time) |
| **11** | Working_hours_gap_mean | Mean deviation time from work time | Mean work deviation time in the most recent week (including the assessment time point) at the assessment time point (only on workdays) |
| **12** | Working_hours_gap_sd | SD of the deviation of working hours | Variation in work deviation time in the most recent week (including the assessment time point) at the time of assessment (only on workdays) |
| **13** | Working_start_gap_mean | Mean deviation time from work start time | Mean time of starting work in the most recent week (including the assessment time point) at the time of assessment (only on workdays) |
| **14** | Working_start_gap_sd | SD of the deviation from starting time of work | Variation in the time since the start of work in the most recent week (including the assessment time point) at the time of assessment (only on workdays) |
| **15** | Working_end_gap_mean | Mean deviation time from work completion time | Mean work completion deviation time in the last week (including the evaluation time point) at the evaluation time point (only on workdays) |
| **16** | Working_end_gap_sd | SD of the deviation time from work completion time | Variation in the time of deviation from work completion in the last week (including the evaluation time point) at the evaluation time point (only on workdays) |
| **17** | Holiday1 | Number of holidays | Days of public holidays in the most recent week (including the assessment time point) at the assessment time point |
| **18** | Holiday2 | Number of days of leave taken | Number of leave days taken in the most recent week (including the evaluation point) of the evaluation point. All types of holidays (regardless of paid or not paid leaves) are included: Paid holidays, accumulated holidays, sick holidays, leave for marriage, maternity leave, etc. |
| **19** | Holiday3 | Number of paid holidays taken by employees | Number of days on paid leave in the most recent week (including the assessment time point) at the assessment time point |
| **20** | Holiday_work | Number of holiday workdays | Number of days working in holiday in the most recent week (including the assessment time point) at the assessment time point |
| **21** | Day_steps_mean | Mean daily step count | Mean daily steps (including 0-step days) in the most recent week (including the time of valuation) at the time of valuation |
| **22** | Day_steps_sd | SD of daily step count | SD of the number of steps per day in the most recent week at the time of valuation (including the date of valuation) (including 0-step days) |
| **23** | Hour_steps_mean_mean | Mean number of steps per hour per day | The mean number of steps per hour per day is calculated for each day, and the mean value for the most recent week (including the assessment time point) of the assessment time point is calculated (including the day of 0 steps) |
| **24** | Hour_steps_mean_sd | SD of the mean number of steps per hour in a day | The mean number of steps per hour per day is calculated for each day, and SD in the most recent week (including the assessment time point) of the assessment time point is calculated (including the day of 0 steps) |
| **25** | Hour_steps_sd_mean | Mean of SD of steps per hour in a day | SD of the number of steps per hour per day is calculated on each day, and the mean value in the most recent week (including the assessment time point) of the assessment time point is calculated (including the day of 0 steps) |
| **26** | Hour_steps_sd_sd | SD of SD of the number of steps per hour in a day | SD of the number of steps per hour per day is calculated on each day, and SD in the most recent week (including the assessment time point) of the assessment time point is calculated (including the day of 0 steps) |
| **27** | Day_calories_mean | Mean daily calories burned | Mean daily calories burned in the most recent week (including the assessment time point) at the assessment time point |
| **28** | Day_calories_sd | SD of daily calories burned | SD of daily calories burned in the most recent week (including the assessment time point) at the assessment time point |
| **29** | Day_distance_mean | Mean moving distance per day | Mean daily distance traveled in the most recent week (including the assessment time point) at the assessment time point |
| **30** | Day_distance_sd | SD of the daily travel distance | SD of the distance traveled per day in the most recent week of the assessment time point (including the assessment time point) |
| **31** | Day_floors_mean | Mean daily climbing and descending floors | Mean number of stairs ascending and descending per day in the most recent week (including the assessment time point) at the assessment time point |
| **32** | Day_floors_sd | SD of the number of ascending and descending floors per day | SD of the number of daily up and down stairs in the most recent week (including the assessment time point) at the assessment time point |
| **33** | Hour_hr_mean_mean | Mean daily heart rate | Mean daily heart rate will be calculated on each day, and the mean in the most recent week (including the assessment time point) at the time of assessment will be calculated |
| **34** | Hour_hr_mean_sd | SD of the mean daily heart rate | Mean daily heart rate is calculated on each day to calculate SD in the most recent week (including the assessment time point) of the assessment time point |
| **35** | Hour_hr_sd_mean | Mean SD of the daily heart rate | SD of the daily heart rate is calculated on each day, and the mean value in the most recent week (including the assessment time point) at the assessment time point is calculated |
| **36** | Hour_hr_sd_sd | SD of SD of the daily heart rate | SD of the daily heart rate is calculated on each day, and SD in the most recent week (including the evaluation time point) at the evaluation time point is calculated |
| **37** | SleepTime_mean | Mean sleep time | Mean sleeping duration (isMainSleep="True) in the most recent week (including the assessment time point) at the assessment time point |
| **38** | SleepTime_sd | SD of sleep duration | SD (isMainSleep="True) of sleeping hours in the most recent week (including the assessment time point) at the assessment time point |
| **39** | Efficiency_mean | Mean sleep efficiency | Mean sleep efficiency (isMainSleep="True) in the most recent week (including the assessment time point) at the assessment time point |
| **40** | Efficiency_sd | SD of sleep efficiency | SD of sleep efficiency (isMainSleep="True) in the most recent week (including the assessment time point) at the assessment time point |
| **41** | SleepStartTime_mean | Mean sleep onset time | Mean sleep onset times (isMainSleep="True) in the most recent week (including the assessment time point) at the assessment time point |
| **42** | SleepStartTime_sd | SD of the sleep onset time | SD (isMainSleep="True) of sleep onset times in the most recent week (including the assessment time point) at the assessment time point |
| **43** | SleepEndTime_mean | Mean sleep termination time | Mean sleep termination times (isMainSleep="True) in the most recent week (including the assessment time point) at the assessment time point |
| **44** | SleepEndTime_sd | SD of the sleep termination time | SD (isMainSleep="True) of sleep termination times in the most recent week (including the assessment time point) at the assessment time point |
| **45** | Private_time_all_mean | Mean leisure time (all days) | Mean leisure time (all days) in the most recent week (including the assessment time point) at the assessment time point |
| **46** | Private_time_all_sd | SD of the leisure time (all days) | SD of leisure time (all days) in the most recent week (including the assessment time point) at the assessment time point |
| **47** | Private_time_workday_mean | Mean leisure time (working days) | Mean leisure time (working days) in the last week (including assessment time point) at the assessment time point |
| **48** | Private_time_workday_sd | SD of the leisure time (working days) | SD of leisure time (working days) in the most recent week (including assessment time point) at the assessment time point |
| **49** | Private_time_holiday_mean | Mean leisure time (holidays) | Mean leisure time (holidays) in the most recent week (including the assessment time point) at the assessment time point |
| **50** | Private_time_holiday_sd | SD of the leisure time (holidays) | SD of leisure time (holidays) in the most recent week (including the assessment time point) at the assessment time point |

SD: standard deviation.
