# Supplementary material for: Predicting Workers’ Stress: Application of a High-Performance Algorithm Using Working-Style Characteristics
Source: JMIR AI. 2024 Aug 2;3:e55840. doi: 10.2196/55840 (PMC11329844; doi:10.2196/55840)
Supplement: Multimedia Appendix 2 [file ai_v3i1e55840_app2.docx]

**Table S2 Multimedia Appendix 2.** Top 10 features with the highest mean feature importance ranking categorized into three levels of teleworking rates using XGBoost (features related to activity [red], work [green], and sleep [blue]).

| **All (n=190)** |  | **Low (n=82)** |  | **Middle (n=69)** |  | **High (n=39)** |  |
| --- | --- | --- | --- | --- | --- | --- | --- |
| Variable | Mean rank | Variable | Mean rank | Variable | mean rank | Variable | Mean rank |
| day_calories_mean | 11.88 | working_start_mean | 10.17 | day_calories_mean | 10.12 | hour_hr_sd_mean | 5.21 |
| working_start_mean | 13.23 | hour_hr_mean_mean | 12.34 | working_start_mean | 11.3 | activity_log_lunch | 7.46 |
| hour_hr_mean_mean | 14.03 | day_calories_mean | 13.51 | working_hours_mean | 15.16 | day_calories_mean | 11.59 |
| hour_hr_sd_mean | 15.2 | sleep_time_sd | 14.29 | hour_hr_sd_mean | 16.13 | hour_hr_mean_mean | 12.92 |
| sleep_start_time_mean | 17.13 | sleep_start_time_mean | 14.87 | efficiency_mean | 16.64 | hour_steps_mean_mean | 15.75 |
| efficiency_mean | 17.43 | activity_log_outgo2 | 16.17 | hour_hr_mean_mean | 16.67 | working_hours_gap_mean | 16.36 |
| sleep_end_time_mean | 18.58 | sleep_end_time_mean | 17.24 | working_end_mean | 17.42 | day_floors_sd | 16.56 |
| working_hours_mean | 18.64 | efficiency_mean | 17.98 | day_steps_mean | 17.69 | sleep_start_time_mean | 16.82 |
| working_end_mean | 19.12 | sleep_time_mean | 18.89 | sleep_time_mean | 18.41 | working_end_mean | 17.41 |
| hour_steps_mean_mean | 19.62 | working_hours_gap_mean | 19.15 | sleep_end_time_mean | 19.12 | efficiency_mean | 17.69 |

sd: standard deviation.
